# *Ab initio *modeling of small proteins by iterative TASSER simulations

**DOI:** 10.1186/1741-7007-5-17

**Published:** 2007-05-08

**Authors:** Sitao Wu, Jeffrey Skolnick, Yang Zhang

**Affiliations:** 1 Center for Bioinformatics and Department of Molecular Bioscience, University of Kansas, 2030 Becker Dr, Lawrence, KS 66047, USA; 2 Center for the Study of Systems Biology, School of Biology, Georgia Institute of Technology, 250 14th Street NW, Atlanta, GA 30318, USA

## Abstract

**Background:**

Predicting 3-dimensional protein structures from amino-acid sequences is an important unsolved problem in computational structural biology. The problem becomes relatively easier if close homologous proteins have been solved, as high-resolution models can be built by aligning target sequences to the solved homologous structures. However, for sequences without similar folds in the Protein Data Bank (PDB) library, the models have to be predicted from scratch. Progress in the *ab initio *structure modeling is slow. The aim of this study was to extend the TASSER (*t*hreading/*asse*mbly/*r*efinement) method for the *ab initio *modeling and examine systemically its ability to fold small single-domain proteins.

**Results:**

We developed I-TASSER by iteratively implementing the TASSER method, which is used in the folding test of three benchmarks of small proteins. First, data on 16 small proteins (< 90 residues) were used to generate I-TASSER models, which had an average C_α_-root mean square deviation (RMSD) of 3.8Å, with 6 of them having a C_α_-RMSD < 2.5Å. The overall result was comparable with the all-atomic ROSETTA simulation, but the central processing unit (CPU) time by I-TASSER was much shorter (150 CPU days vs. 5 CPU hours). Second, data on 20 small proteins (< 120 residues) were used. I-TASSER folded four of them with a C_α_-RMSD < 2.5Å. The average C_α_-RMSD of the I-TASSER models was 3.9Å, whereas it was 5.9Å using TOUCHSTONE-II software. Finally, 20 non-homologous small proteins (< 120 residues) were taken from the PDB library. An average C_α_-RMSD of 3.9Å was obtained for the third benchmark, with seven cases having a C_α_-RMSD < 2.5Å.

**Conclusion:**

Our simulation results show that I-TASSER can consistently predict the correct folds and sometimes high-resolution models for small single-domain proteins. Compared with other *ab initio *modeling methods such as ROSETTA and TOUCHSTONE II, the average performance of I-TASSER is either much better or is similar within a lower computational time. These data, together with the significant performance of automated I-TASSER server (the Zhang-Server) in the 'free modeling' section of the recent Critical Assessment of Structure Prediction (CASP)7 experiment, demonstrate new progresses in automated *ab initio *model generation. The I-TASSER server is freely available for academic users .

## Background

Prediction of protein structure from amino-acid sequences has been one of the most challenging problems in computational structural biology for many years [1,2]. Historically, protein structure prediction was classified into three categories: (i) comparative modeling [3,4], (ii) threading [5-9], and (iii) *ab initio *folding [10-15]. The first two approaches build protein models by aligning query sequences onto solved template structures. When close templates are identified, high-resolution models could be built by the template-based methods. If templates are absent from the Protein Data Bank (PDB) library, the models need to be built from scratch, i.e. *ab initio *folding. This is the most difficult category of protein-structure prediction [16,17].

With increasing protein sizes, the conformational phase space of sampling also sharply increases, which makes the *ab initio *modeling of larger proteins extremely difficult [18]. Current *ab initio *predictions are mainly focused on small proteins. Several successful examples have been reported in literature. For example, based on an *ab initio *approach designed to globally optimize their potential energy function, Liwo et al were able to build models of C_α _root mean square deviation (RMSD) to native < 6Å for protein fragments of up to 61 residues [10]. Using the ROSETTA program [11], Simon et al reported 73 successful structure predictions out of 172 target proteins with lengths of < 150 residues, with C_α_-RMSD < 7Å in the top five models [19]. Using TOUCHSTONE-II software, Zhang et al reported 83 foldable cases from 125 target proteins (up to 174 residues) with C_α_-RMSD < 6.5Å in the top five models [12]. Recently, Bradley et al demonstrated an exciting achievement by building several high-resolution models for proteins of < 90 residues [13]. By combining low-resolution and high-resolution sampling, the authors used the all-atomic ROSETTA to predict high-resolution models with C_α_-RMSD < 1.5Å for 5 of 16 small proteins. The average C_α_-RMSD for all the 16 proteins was 3.8Å in the best of the top five clusters. The CPU time cost, however, is expensive and ~150 CPU days are required for the all-atom sampling of each target.

In this work, we aimed to investigate the possibility of generating high-resolution models of small proteins in an automated and fast simulation. We developed a new method, I-TASSER, which implements TASSER [18,20] in an iterative mode and also exploits new force-field optimization and fragment identification. We tested the I-TASSER method on three independent benchmark sets. The result shows that I-TASSER has a comparable overall performance with the all-atomic ROSETTA but with far lower CPU cost. It also demonstrates that I-TASSER clearly outperforms the TOUCHSTONE-II method.

## Results and discussion

We tested the folding performance of I-TASSER on small proteins. To avoid contamination with homologous proteins, any template with > 20% sequence identity to the target sequence was removed from our template library. Moreover, if a template could be detected by the Position Specific Iterative (PSI)-BLAST program with an E-value < 0.05, it would also be excluded. We note that the homology exclusion cutoff used here is more stringent than that used by Bradley et al [13], who only excluded templates with a PSI-BLAST E-value < 0.05 but without sequence identity cutoff, and that used by Zhang et al [12], who only excluded the templates with sequence identity > 30% but without PSI-BLAST checking. In the sense that all homologous templates had been completely excluded, we termed the corresponding simulations "*ab initio*" modeling, following the notation by others [10,12,13,21].

For the evaluation of the predicted models, we used both the RMSD and TM-score [22]. Although RMSD can give an explicit concept of modeling errors, in some cases, a local error (e.g., tail misorientation) can cause a large RMSD value even though the global topology is correct. TM-score is defined as [22].

TM-score=1N∑i=1Nali11+(di/d0)2,
 MathType@MTEF@5@5@+=feaafiart1ev1aaatCvAUfKttLearuWrP9MDH5MBPbIqV92AaeXatLxBI9gBaebbnrfifHhDYfgasaacH8akY=wiFfYdH8Gipec8Eeeu0xXdbba9frFj0=OqFfea0dXdd9vqai=hGuQ8kuc9pgc9s8qqaq=dirpe0xb9q8qiLsFr0=vr0=vr0dc8meaabaqaciaacaGaaeqabaqabeGadaaakeaacqqGubavcqqGnbqtcqqGTaqlcqqGZbWCcqqGJbWycqqGVbWBcqqGYbGCcqqGLbqzcqGH9aqpdaWcaaqaaiabigdaXaqaaiabd6eaobaadaaeWbqaamaalaaabaGaeGymaedabaGaeGymaeJaey4kaSYaaeWaaeaadaWcgaqaaiabdsgaKnaaBaaaleaacqWGPbqAaeqaaaGcbaGaemizaq2aaSbaaSqaaiabicdaWaqabaaaaaGccaGLOaGaayzkaaWaaWbaaSqabeaacqaIYaGmaaaaaaqaaiabdMgaPjabg2da9iabigdaXaqaaiabd6eaonaaBaaameaacqWGHbqycqWGSbaBcqWGPbqAaeqaaaqdcqGHris5aOGaeiilaWcaaa@5095@

where *N *is the number of residues of the query sequence and *N*_*ali *_is the number of aligned residues in a threading alignment. For a full-length model, *N *and *N*_*ali *_are identical. *d*_*i *_is the distance of the *i*th C_α _pair between model and native after superposition, and d0=1.24N−153−1.8
 MathType@MTEF@5@5@+=feaafiart1ev1aaatCvAUfKttLearuWrP9MDH5MBPbIqV92AaeXatLxBI9gBaebbnrfifHhDYfgasaacH8akY=wiFfYdH8Gipec8Eeeu0xXdbba9frFj0=OqFfea0dXdd9vqai=hGuQ8kuc9pgc9s8qqaq=dirpe0xb9q8qiLsFr0=vr0=vr0dc8meaabaqaciaacaGaaeqabaqabeGadaaakeaacqWGKbazdaWgaaWcbaGaeGimaadabeaakiabg2da9iabigdaXiabc6caUiabikdaYiabisda0maakeaabaGaemOta4KaeyOeI0IaeGymaeJaeGynaudaleaacqaIZaWmaaGccqGHsislcqaIXaqmcqGGUaGlcqaI4aaoaaa@3CB5@. As TM-score weights small distances stronger than larger distances, it is more sensitive to global topology than is RMSD. According to Zhang and Skolnick [22], TM-score = 1 indicates two identical structures and TM-score < 0.17 indicates random structure pairs. A TM-score of > 0.5 means two structures with the same folding.

### Benchmark I: 16 proteins from the data of Bradley et al

Table 1 shows the modeling result of I-TASSER on 16 small proteins that were used by Bradley et al [13]. This benchmark set includes 3 α proteins, 2 β proteins, and 11 αβ proteins with pairwise sequence identity < 30%. If we define a high-resolution model as that with C_α_-RMSD to native ≤ 1.5Å, I-TASSER predicts high-resolution models for one target '1ogwA' (see Figure 2A for the model superimposed on the native structure). For the best of the top five clusters, most of the targets (12/16) had a medium resolution, with a C_α_-RMSD of 1.5–5Å. For the remaining three targets, I-TASSER could not correctly fold the proteins. One of them (1tif_) has a long swinging tail at the C-terminal. For the other two (1dcjA_ and 1o2fB_), both having a topology of four parallel β-strands flanked by two α-helices, the imperfection of the I-TASSER force field is obviously responsible for the failure because the energy of the native structures is higher than that of the largest clusters.

For the first predicted model of the highest cluster density, the overall average C_α_-RMSD for the 16 target proteins was 4.3Å with average TM-score of 0.59. If we consider the best model in the top five predictions, the average C_α_-RMSD to the native is 3.8Å and TM-score was 0.61. Figure 2(b,c) shows typical examples of both medium-resolution and low-resolution predicted models.

As a comparison, the table also lists the all-atomic ROSSETA predictions for the 16 proteins (columns 4–6). ROSETTA predicted more high-resolution models than I-TASSER does. ROSETTA had three models < 1.5Å in round 1, four models in round 2, and two models in the top five clusters. The difference in the number of high-resolution models may indicate the resolution limitation of the reduced potential used in I-TASSER modeling. However, ROSETTA had more low-resolution models than did I-TASSER. If we define low-resolution models as those with a C_α_-RMSD > 5Å, ROSETTA had seven low-resolution models in round 1, five low-resolution models in round 2, and four low-resolution models in the best of the top five clusters. I-TASSER had only three low-resolution models in the best of the top five clusters. The overall average C_α_-RMSD of the best of the top five I-TASSER models is 3.8Å, comparable with that of ROSETTA (round 1: 5.1Å; round 2: 4.7Å; top five: 3.8Å). The statistical equivalency of these two methods was at the 5% significance level under the Wilcoxon rank sum test based on C_α_-RMSD. However, the CPU time cost by I-TASSER was much shorter (~5 CPU hours vs. 150 CPU days). The main reason for the CPU saving might be that I-TASSER operates under reduced modeling, whereas the ROSETTA modeling is at an atomic level. The simulations on multiple homologous sequences also increase the computing time for ROSETTA [13].

Figure 3A shows the plot of C_α_-RMSD to native of the best model in the top five clusters versus that of the best threading alignments over the same aligned regions (star symbols). Almost all the final models (except 1b72A) were much closer to native than the best threading alignments, as indicated by the reduction of RMSD values. Along the same aligned region, the average C_α_-RMSD for the models and the templates were 3.6Å and 5.7Å respectively. The significant improvement of I-TASSER models on the threading alignments were also found here (Figure 3B), where the average TM-score for the models and the template were 0.61 and 0.49 respectively. Again, most of final models had a higher TM-score than that of the best threading alignments (a list of the best templates with the highest TM-score for each target protein in this study is available online at ).

### Benchmark II: 20 proteins from Zhang et al

In this benchmark set, we took 20 proteins from the data of Zhang et al [12]: six α-proteins, six β-proteins, and eight αβ-proteins, with sizes ranging from 47 to 118 residues. These 20 proteins were selected so that they and the proteins used in benchmark I had pairwise sequence identity of < 30%.

As shown in Table 2, in this benchmark set, most of the targets had medium resolution, with C_α_-RMSD to native of 1.5–5Å by I-TASSER. More specifically, for the best of the top five clusters, 14 targets had medium resolution, 5 targets had low resolution, and 1 target (1cy5A) had high resolution. Typical examples from the three categories are shown in Figure 2(D–F). The comparisons of the final models with the initial threading alignments are shown in Figure 3 (A,B; circles). Again, the global topology of the final models was significantly closer to the native structure than were the threading alignments. The average C_α_-RMSD and TM-score of the initial threading alignments were 6.1Å and 0.53 respectively, but after I-TASSER simulations, they improved to 3.4Å and 0.65.

Compared with the TOUCHSTONE-II modeling [12], I-TASSER predicted better models in 17 cases, with lower C_α_-RMSD in the best of the top 5 clusters. Only in three cases did I-TASSER models have slightly higher C_α_-RMSD, i.e. 1bq9A (5.0Å vs. 4.8Å), 256bA (3.4Å vs. 3.2Å), and 2pcy_ (4.6Å vs. 4.3Å). The overall average C_α_-RMSD results for the TOUCHSTONE-II and I-TASSER models were 5.9Å and 3.9Å respectively. Statistically, the result of I-TASSER was better than that of TOUCHSTONE-II at the 1% significance level using the Wilcoxon rank sum test.

The algorithm of TOUCHSTONE-II was previously developed in our group, and searches for protein conformations on a cubic lattice system. Each residue in TOUCHSTONE-II is represented by its C_α_, C_β_, and the side-chain center of mass (the CABS model [12]). The force field consists of a variety of knowledge-based statistical potentials from PDB and the side-chain contact restraints predicted by PROSPECTOR_3 [9]. In TASSER [20], we assemble the protein models directly using the continuous fragments from the PROSPECTOR_3 alignment, in which the conformations are searched in an "on-and-off" lattice system, i.e. the threading-aligned regions are modeled off-lattice and the unaligned loop regions on-lattice. Each residue is represented by the C_α _and the side-chain center of mass (the CAS model [20]). The force field was developed from TOUCHSTONE-II, with new potentials including the sequence-specified pair-wise potential [23], C_α _distance correlations from sequence-specific fragments [20], and the more accurate secondary structure-specified hydrogen bonding [24]. The force field of I-TASSER is mainly developed from that of TOUCHSTONE-II and TASSER. The new components in I-TASSER include: (i) the new neural network hydrophobic potential as described in equation 3 in the Methods section and the decoy-based reparameterization of all weight-factors based on target categories; (ii) the development of the new PPA threading program, which provides different assembly fragments and restraints; and (iii) the two-step iterative refinement simulations. For conformational sampling, the introduction of the on-and-off lattice fragment assembly simulation in TASSER and I-TASSER is the key factor to speed up the search of important phase spaces because the usage of rigid-body fragments dramatically reduces the entropy of the searched space. The modeling improvement data shown in Table 3 demonstrates the progress of I-TASSER in both potentials and sampling since TOUCHSTONE-II [12].

### Benchmark III: 20 non-homologous small proteins selected from the PDB

For the testing of the generality of I-TASSER folding on small proteins, we constructed the third benchmark proteins directly from the PDB library. As listed in Table 3, this set includes seven α-proteins, six β-proteins, and seven αβ-proteins, with lengths ranging from 56 to 118 residues. To avoid the redundancy of the benchmarks, the proteins were selected so that this set and the previously used 36 target proteins had sequence identity between all the pairs of < 30%. The proteins were randomly taken from PDB, but the targets with unusual topology (such as coiled-coil or a structure with a long tail) were excluded.

The average C_α_-RMSD of the best in top five models by I-TASSER was 3.9Å (4.8Å for rank 1 models), which was similar to that of benchmarks I and II. Again, there was one model (1cqkA) with a high-resolution prediction, as presented in Figure 2g. There were 14 medium-resolution predictions and 5 low-resolution ones. The typical examples from these two categories are shown in Figure 2(H and I). The global topology of the final models was also markedly closer to the native structure than the threading alignments, as shown in Figure 3 (triangle symbols). Overall, the model quality and the C_α_-RMSD distribution in this independent set was comparable with the benchmark sets taken from Bradley et al [13] and Zhang et al [12], which demonstrates the robustness and stability of the I-TASSER modeling on *ab initio *small-protein folding. The I-TASSER method was also tested in recent blind CASP7 experiments [25], where the overall TM-score of the I-TASSER models was significantly better than that of all other automated methods (>5% higher than the second-best CASP7 server).

## Conclusion

In summary, we have developed a new approach to protein structure modeling by iteratively implementing the TASSER method. Meanwhile, we have introduced a new profile-profile alignment approach for the I-TASSER fragment collection, and a new neural network-trained hydrophobic potential, which has been implemented in a reduced Monte Carlo simulation for the first time.

The benchmark proteins were taken from three independent sources, in which any solved structure that had a sequence identity of > 20% to the targets and could be detected by PSI-BLAST with an E-value of < 0.05 was removed from the template library.

The I-TASSER folding showed comparable overall results with the all-atomic ROSETTA simulation, especially in the medium-resolution region. It is noteworthy that, even with reduced modeling, the current I-TASSER has the capacity to generate high-resolution models, although the frequency of high-resolution cases was lower than that of the all-atomic ROSETTA. Further development of the atomic potential for the I-TASSER might be helpful in increasing the modeling accuracy in the high-resolution region, but it would certainly increase the CPU cost of I-TASSER. Currently, the average CPU time for small proteins is about 5 CPU hours for I-TASSER, whereas the CPU cost for the atomic ROSETTA modeling is 150 CPU days per target.

The I-TASSER modeling results obviously outperform those generated by TOUCHSTONE-II [12], with the average C_α_-RMSD reducing from 5.9Å to 3.9Å for the same protein set. As the sequence identity cutoff used here was more stringent than that used by TOUCHSTONE-II, the improvement demonstrates the progress of I-TASSER in both force field and conformational sampling.

Although the benchmark proteins were taken from different sources, the overall performance of I-TASSER was very similar. For the first predicted models, the average C_α_-RMSD ranged from 4.3Å to 4.8Å and the average TM-score ranged from 0.59 to 0.64 for the three benchmarks. For the best models in the top five predictions, the average C_α_-RMSD ranged from 3.8Å to 3.9Å and the average TM-score ranged from 0.61 to 0.65. This modeling stability, along with the consistent results from I-TASSER server in the "free modeling" section of the recent CASP7 experiment, demonstrates the robustness of I-TASSER method in predicting correct folds for small proteins. Meanwhile, the capacity of generating medium-resolution to high-resolution models using reduced modeling represents new progress in the field of *ab initio *modeling.

## Methods

**Figure 1:**
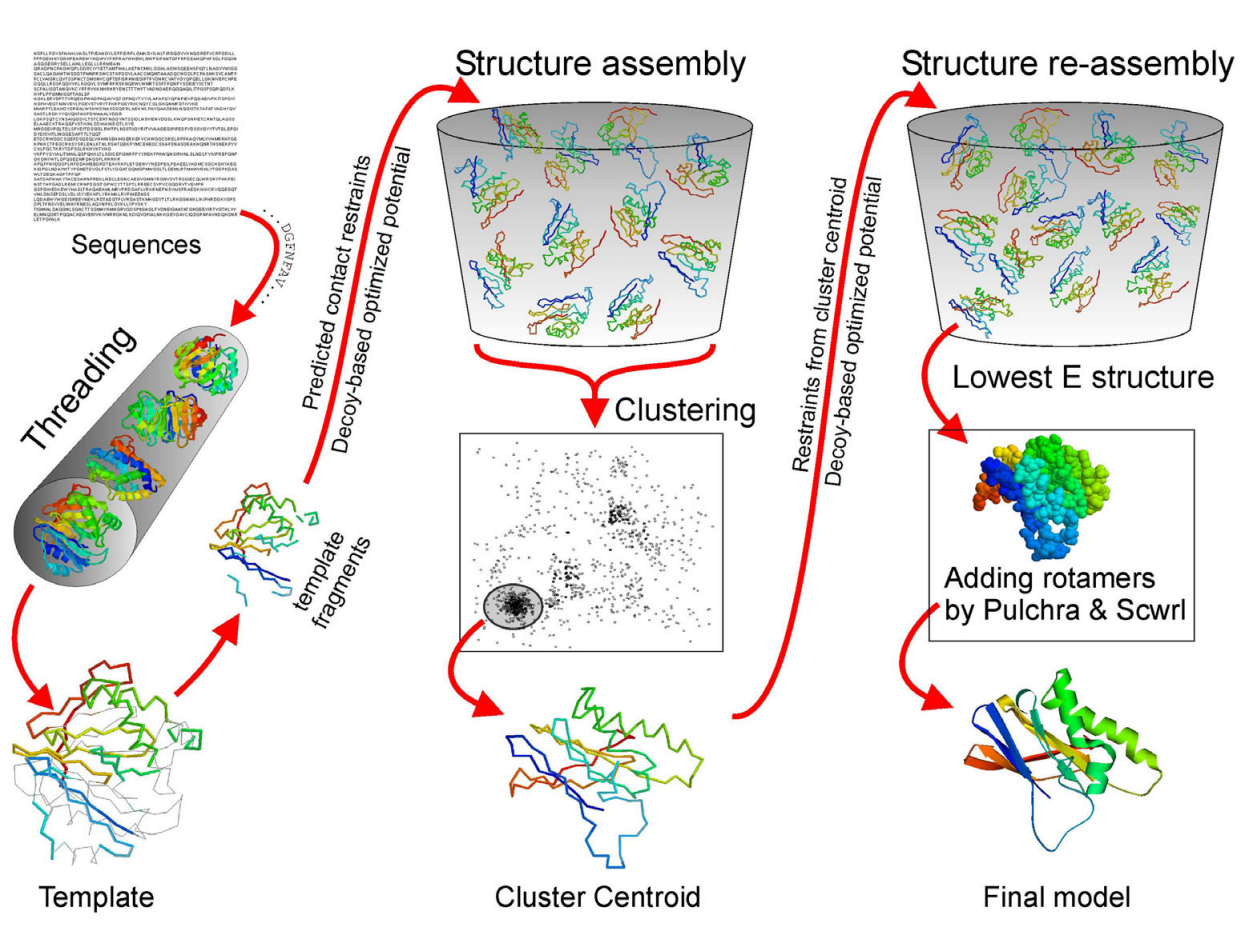
Flowchart of I-TASSER method for protein structure prediction.

**Figure 2:**
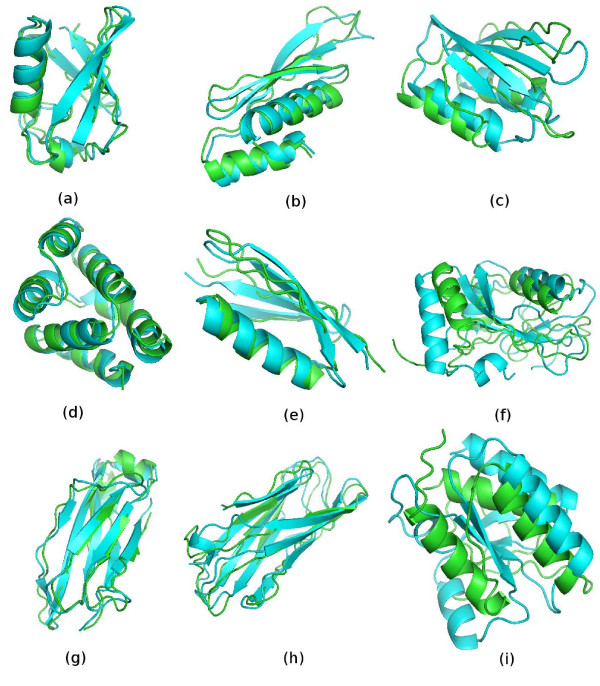
**Examples of I-TASSER models from three independent benchmark sets**. The green color is for I-TASSER models and blue for the native structures. (A–C) are from benchmark I (Bradley et al [13]); (D–F) are from benchmark II (Zhang et al [12]); and (G–I) are from benchmark III, selected directly from the PDB library. Column 1 contains the high-resolution models with a Cα-RMSD ≤ 1.5Å; column 2 contains the medium-resolution models with a Cα-RMSD of 1.5–5Å; column 3 contains the low-resolution models with a Cα-RMSD > 5Å. The Cα-RMSD value for the examples are: **(A) **1ogwA_ (1.1Å), **(B) **1di2A_ (2.3Å), **(C) **1dcjA_(10.0Å), **(D) **1cy5A (1.5Å), **(E) **1pgx (3.1Å), **(F) **1gnuA (8.2Å), **(G) **1cqkA (1.5Å), **(H) **1gyvA (3.3Å), **(I) **1no5A(10.5Å). The pictures were generated using PyMOL software [45].

**Figure 3:**
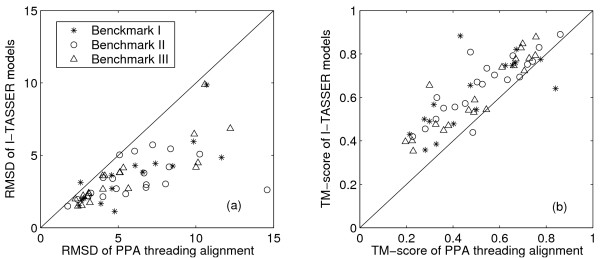
**Comparison of I-TASSER models with the PPA threading alignment results**. **(A) **Cα-RMSD to native of the I-TASSER models versus Cα-RMSD to native of the best threading alignment over the same aligned regions. **(B) **TM-score of the I-TASSER models versus TM-score of the best threading alignments.

The I-TASSER method is an extension of the previous TASSER (*t*hreading/*asse*mbly/*r*efinement) method [18,20]. The overall procedure is described in Figure 1. This method has also been used in the automated server section, named 'Zhang-Server', in the recent CASP7 experiment.

### PPA threading

The query sequence is first threaded through the PDB library [26] to identify appropriate local fragments, which will be adopted for further structural reassembly. The threading method used in I-TASSER is a simple profile-profile alignment (PPA) approach. The alignment score between the *i*th residue of the query sequence and the *j*th residue of the template structure is defined as

Score(i,j)=∑k=120Pquery(i,k)Ltemplate(j,k)+c1δ(squery(i),stemplate(j))+c2,
 MathType@MTEF@5@5@+=feaafiart1ev1aaatCvAUfKttLearuWrP9MDH5MBPbIqV92AaeXatLxBI9gBaebbnrfifHhDYfgasaacH8akY=wiFfYdH8Gipec8Eeeu0xXdbba9frFj0=OqFfea0dXdd9vqai=hGuQ8kuc9pgc9s8qqaq=dirpe0xb9q8qiLsFr0=vr0=vr0dc8meaabaqaciaacaGaaeqabaqabeGadaaakeaacqWGtbWucqWGJbWycqWGVbWBcqWGYbGCcqWGLbqzcqGGOaakcqWGPbqAcqGGSaalcqWGQbGAcqGGPaqkcqGH9aqpdaaeWaqaaiabdcfaqnaaBaaaleaacqWGXbqCcqWG1bqDcqWGLbqzcqWGYbGCcqWG5bqEaeqaaOGaeiikaGIaemyAaKMaeiilaWIaem4AaSMaeiykaKIaemitaW0aaSbaaSqaaiabdsha0jabdwgaLjabd2gaTjabdchaWjabdYgaSjabdggaHjabdsha0jabdwgaLbqabaGccqGGOaakcqWGQbGAcqGGSaalcqWGRbWAcqGGPaqkaSqaaiabdUgaRjabg2da9iabigdaXaqaaiabikdaYiabicdaWaqdcqGHris5aOGaey4kaSIaem4yam2aaSbaaSqaaiabigdaXaqabaacciGccqWF0oazdaqadaqaaiabdohaZnaaBaaaleaacqWGXbqCcqWG1bqDcqWGLbqzcqWGYbGCcqWG5bqEaeqaaOGaeiikaGIaemyAaKMaeiykaKIaeiilaWIaem4Cam3aaSbaaSqaaiabdsha0jabdwgaLjabd2gaTjabdchaWjabdYgaSjabdggaHjabdsha0jabdwgaLbqabaGccqGGOaakcqWGQbGAcqGGPaqkaiaawIcacaGLPaaacqGHRaWkcqWGJbWydaWgaaWcbaGaeGOmaidabeaakiabcYcaSaaa@876F@

where *P*_*query*_(*i*, *k*) is the frequency of the *k*th amino acid at the *i*th position of the query sequence when a PSI-BLAST [27] search of the query sequence runs against a non-redundant sequence database  and   . with an E-value cutoff of 0.001; *L*_*template*_(*j*, *k*) is the log-odds profile of template sequence in the PSI-BLAST search; *S*_*query*_(*i*) is the secondary structure prediction from PSIPRED [28] for the *i*th residue of the query sequence and *S*_*template*_(*j*) the secondary structure assignment by DSSP [29] for the *j*th residue of the template. The weight factor *c*_1 _is an adjustable parameter for balancing the profile term and the secondary structure matches; the shift constant *c*_2 _is introduced to avoid the alignment of unrelated regions in the local alignment [8]. The Needleman-Wunsch dynamic programming algorithm [30] is used to find the best match between query and template sequences. A position-dependent gap penalty is used: no gap is allowed inside the secondary structure regions; gap opening (*c*_3_) and gap extension (*c*_4_) penalties apply to other regions; and the ending gap penalty is ignored. The best tuning parameters, based on our trial and error on the ProSup benchmark [31], are: *c*_1 _= 0.6, *c*_2 _= 1.0, *c*_3 _= 7.0, *c*_4 _= 0.5.

### Structure assembly

A protein chain in the I-TASSER modeling is divided into aligned and unaligned regions based on the PPA alignment, where the aligned regions are modeled off-lattice for maximum accuracy of the secondary structure blocks and the unaligned regions are simulated on a cubic lattice system for computational efficiency [20].

For a given alignment, an initial full-length model is built up by connecting the continuous secondary structure fragments (≥ 5 residues) through a random walk of C_α_-C_α _bond vectors of variable lengths from 3.26 to 4.35Å. Only excluded volume and geometric constraints of virtual C_α_-C_α _bond angles (65–165°) are considered during the initial model-building procedure. The side-chain center of mass is determined by a two-rotamer approximation that depends on whether the local backbone configuration is extended or compact. To guarantee that the last step of this random walk can quickly arrive at the first C_α _of the next template fragment, the distance *l *between the current C_α _and the first C_α _of the next template fragment is checked at each step of the random walk, and only walks with *l *< 3.54*n *are allowed, where *n *is the number of remaining C_α_-C_α _bonds in the walk. If a template gap is too big to span by a specified number of unaligned residues, a big C_α_-C_α _bond will remain at the end of the random walk and a spring-like force that acts to draw sequential fragments close will be applied in subsequent Monte Carlo simulations until a physically reasonable bond length is achieved.

The initial full-length models are submitted to parallel-exchange Monte Carlo sampling [32] for assembly/refinement. Two kinds of conformational updates (off-lattice and on-lattice) are implemented. (i) Off-lattice movements of the aligned regions involve rigid fragment translations and rotations that are controlled by the three Euler angles. The fragment length normalizes the movement amplitude so that the acceptance rate is approximately constant for fragments with different sizes. (ii) The lattice-confined residues are subjected to 2–6 bond movements and multi-bond sequence shifts [12]. Overall, the tertiary topology varies by the rearrangement of the continuously aligned substructures, where the local conformation of the off-lattice substructures remains unchanged during assembly.

### Force field

The inherent I-TASSER assembly force field is similar to TASSER, which includes predicted secondary structure propensities from PSIPRED [28], backbone hydrogen bonds [24], and a variety of statistical short-range and long-range correlations [12,18,20]. The major new potential in I-TASSER is the incorporation of the predicted accessible surface area (ASA) through the neural network (NN) [33].

For the purpose of fast calculations of the ASA effect, the hydrophobic energy in I-TASSER is defined by

EASA=−∑i(xi2x02+yi2y02+zi2z02−2.5)*P(i),
 MathType@MTEF@5@5@+=feaafiart1ev1aaatCvAUfKttLearuWrP9MDH5MBPbIqV92AaeXatLxBI9gBaebbnrfifHhDYfgasaacH8akY=wiFfYdH8Gipec8Eeeu0xXdbba9frFj0=OqFfea0dXdd9vqai=hGuQ8kuc9pgc9s8qqaq=dirpe0xb9q8qiLsFr0=vr0=vr0dc8meaabaqaciaacaGaaeqabaqabeGadaaakeaacqWGfbqrdaWgaaWcbaGaemyqaeKaem4uamLaemyqaeeabeaakiabg2da9iabgkHiTmaaqafabaWaaeWaaeaadaWcaaqaaiabdIha4naaDaaaleaacqWGPbqAaeaacqaIYaGmaaaakeaacqWG4baEdaqhaaWcbaGaeGimaadabaGaeGOmaidaaaaakiabgUcaRmaalaaabaGaemyEaK3aa0baaSqaaiabdMgaPbqaaiabikdaYaaaaOqaaiabdMha5naaDaaaleaacqaIWaamaeaacqaIYaGmaaaaaOGaey4kaSYaaSaaaeaacqWG6bGEdaqhaaWcbaGaemyAaKgabaGaeGOmaidaaaGcbaGaemOEaO3aa0baaSqaaiabicdaWaqaaiabikdaYaaaaaGccqGHsislcqaIYaGmcqGGUaGlcqaI1aqnaiaawIcacaGLPaaaaSqaaiabdMgaPbqab0GaeyyeIuoakiabcQcaQiabdcfaqjabcIcaOiabdMgaPjabcMcaPiabcYcaSaaa@5A92@

where (*x*_*i*_, *y*_*i*_, *z*_*i*_) is the coordinate of *i*th residue at the ellipsoid Cartesian system of the given protein conformation and (*x*_0_, *y*_0_, *z*_0_) is the principle axes length. Thus, 2.5 is a suitable parameter to tune the average depth of the exposed residues. The two-state (expose/bury) neural network was trained on 3365 non-redundant high-resolution protein structures on the basis of their sequence profile from PSI-BLAST [27]. The maximum ASA value in an extended tripeptide (Ala-X-Ala) is taken from Ahmad et al [34]. Twelve different ASA fraction cutoffs (0.05, 0.1, ... 0.6) are used to define the residue expose status in the NN training. The residue expose index is P(i)=∑j=112aj
 MathType@MTEF@5@5@+=feaafiart1ev1aqatCvAUfKttLearuWrP9MDH5MBPbIqV92AaeXatLxBI9gBaebbnrfifHhDYfgasaacH8akY=wiFfYdH8Gipec8Eeeu0xXdbba9frFj0=OqFfea0dXdd9vqai=hGuQ8kuc9pgc9s8qqaq=dirpe0xb9q8qiLsFr0=vr0=vr0dc8meaabaqaciaacaGaaeqabaqabeGadaaakeaacqWGqbaucqGGOaakcqWGPbqAcqGGPaqkcqGH9aqpdaaeWaqaaiabdggaHnaaBaaaleaacqWGQbGAaeqaaaqaaiabdQgaQjabg2da9iabigdaXaqaaiabigdaXiabikdaYaqdcqGHris5aaaa@3BE9@, where *a*_*j *_is the two-state neural network prediction of exposure (*a*_*j *_= 1) or burial (*a*_*j *_= -1) with the *j*th ASA fraction cutoff, which has a strong correlation with the real value of ASA. For an independent set of 2234 non-homologous proteins used by Zhang and Skolnick [18,20], the overall correlation coefficient between the predicted *P*(*i*) and the real exposed area assigned by STRIDE [35] is 0.71, whereas the same correlation for the widely-used Hopp-Woods [36] and Kyte-Doolittle [37] hydrophobicity indices are 0.42 and 0.39 respectively. One of the reasons for the higher correlation is that NN prediction explores the sequence-profile information, whereas the Hopp-Woods and Kyte-Doolittle parameters are sequence-independent.

### Second-round TASSER simulation

The structure trajectories of the first-round TASSER simulations are clustered by SPICKER [38]. The cluster centroids are obtained by averaging all the clustered structures after superposition, which generally have substantial steric clashes and can be over-compressed [39]. Following the clustering, the TASSER Monte Carlo simulation is implemented again, and this starts from the cluster centroid conformations (see Figure 1). The distance and contact restraints in the second-round TASSER are taken from the combination of the centroid structures and the PDB structures searched by the structure alignment program TM-align [40] based on the cluster centroids. The conformation with the lowest energy in the second round is selected. Finally, Pulchra [41] is used to add backbone atoms (N, C, O) and Scwrl_3.0 [42] is used to build side-chain rotamers. The sidechain-building procedure by Pulchra and Scwrl does not modify the C_α _coordinates.

At this point, one of the main purposes of the second-round TASSER is to remove the steric clashes of the cluster centroids. Based on a benchmark test of 200 proteins < 300 residues in size (unpublished data), after the second round of TASSER, the average number of steric clashes for the first cluster reduces from 79 to 0.8. Here, a clash is defined as a pair of residues with C_α _distance < 3.6Å [43]. For the PDB experimental structures, the average number of steric clashes for the 200 proteins is 0.46, which is close to that of the second-round TASSER models. However, as strong distance and contact restraints have been implemented in the second-round simulation, the topology improvement of the models is modest. For the 200 test proteins, the average TM-score [22] increases from 0.5734 to 0.5801 (1.2%) and C_α_-RMSD to native decreases from 6.67Å to 6.52Å compared with the cluster centroid of the first round. Another option of removing the steric clashes is simply to use a TASSER decoy closest to the cluster centroid, but in that case, the average TM-score decreases to 0.5583 (by 2.7%) and C_α_-RMSD to native increases to 7.15Å.

We also tried MODELLER [3] and NEST [44] softwares to refine the centroid models. In both cases, the average C_α_-RMSD was increased in comparison with the cluster centroids. In particular, these tools cannot entirely remove the steric clashes. For the 200 test cases, the average numbers of remaining steric clashes of MODELLER and NEST models were 16.7 and 22.6 respectively.

## Authors' contributions

SW, JS, and YZ developed the I-TASSER method and drafted the paper. SW collected benchmark protein sets, carried out I-TASSER simulations, and performed data analysis. All authors read and approved the final manuscript.

## Figures and Tables

**Table 1:** Summary of I-TASSER modeling on benchmark I in comparison with atomic ROSETTA [13]

			C_α_-RMSD (Å) of ROSETTA models			C_α_-RMSD (Å) (TM-score) of I-TASSER models	
			
Protein name	Length (residues)	Secondary structure	Round 1	Round 2	Best in top five clusters	First cluster	Best in top five clusters
1b72A	49	α	0.8	1.1	1.0	3.3 (0.64)	3.1 (0.64)
1shfA	59	β	11.1	10.8	10.9	1.7 (0.75)	1.7 (0.75)
1tif_	59	αβ	5.3	4.1	3.8	7.0 (0.33)	7.0 (0.36)
2reb_2	60	αβ	1.2	2.1	1.3	5.6 (0.37)	4.7 (0.57)
1r69_	61	α	2.1	1.2	1.7	1.9 (0.75)	1.9 (0.75)
1csp_	67	β	5.1	4.7	5.1	2.1 (0.76)	2.1 (0.76)
1di2A_	69	αβ	2.6	2.6	1.9	2.3 (0.78)	2.3 (0.78)
1n0uA4	69	αβ	9.9	10.2	2.7	4.4 (0.48)	4.4 (0.48)
1mla_2	70	αβ	8.4	8.7	7.2	2.8 (0.66)	2.7 (0.66)
1af7__	72	α	10.1	10.4	1.7	4.2 (0.49)	4.2 (0.49)
1ogwA_	72	αβ	2.7	1.0	2.6	1.1 (0.88)	1.1 (0.88)
1dcjA_	73	αβ	3.2	2.5	2.0	10.5 (0.39)	10.0 (0.39)
1dtjA_	74	αβ	1.0	1.2	1.8	1.9 (0.80)	1.7 (0.82)
1o2fB_	77	αβ	10.1	N/A	10.3	7.1 (0.41)	5.2 (0.43)
1mkyA3	81	αβ	3.2	6.3	3.7	5.2 (0.40)	4.5 (0.50)
1tig_	88	αβ	4.1	3.5	2.4	7.7 (0.50)	4.4 (0.54)
Average	69		5.1	4.7	3.8	4.3 (0.59)	3.8 (0.61)

**Table 2:** Summary of I-TASSER modeling on benchmark II in comparison with TOUCHSTONE-II [12]

			C_α_-RMSD (Å) of TOUCHSTONE-II models	C_α_-RMSD (Å) (TM-score) of I-TASSER models	
			
Protein name	Length (residues)	Secondary structure	Best in top five clusters	First cluster	Best in top five clusters
1gpt_	47	αβ	4.0	5.2 (0.54)	3.8 (0.56)
1tfi_	47	β	6.2	4.6 (0.56)	4.0 (0.57)
1bq9A	53	β	4.8	7.3 (0.41)	5.0 (0.46)
1pgx_	59	αβ	6.0	3.1 (0.55)	3.1 (0.55)
1ah9_	63	β	5.1	4.3 (0.56)	2.8 (0.67)
1aoy_	65	α	4.7	4.5 (0.70)	2.7 (0.70)
1sro_	71	β	4.3	3.4 (0.66)	3.0 (0.68)
1kjs_	74	α	8.2	8.5 (0.38)	5.7 (0.50)
1vcc_	76	αβ	7.3	5.7 (0.44)	5.7 (0.44)
1npsA	88	αβ	3.4	2.1 (0.79)	2.1 (0.79)
1hbkA	89	α	8.5	3.5 (0.69)	3.5 (0.69)
1cy5A	92	α	1.8	1.5 (0.89)	1.5 (0.89)
1bm8_	99	αβ	9.0	6.3 (0.42)	6.3 (0.42)
2pcy_	99	β	4.3	4.6 (0.66)	4.6 (0.66)
256bA	106	α	3.2	3.4 (0.77)	3.4 (0.77)
1cewI	108	αβ	6.3	3.6 (0.73)	3.6 (0.73)
1thx_	108	αβ	2.3	2.1 (0.83)	2.1 (0.83)
1sfp_	111	β	6.0	5.1 (0.75)	5.1 (0.75)
1gnuA	117	αβ	9.3	8.2 (0.58)	6.5 (0.60)
2a0b_	118	α	12.8	2.5 (0.81)	2.5 (0.81)
Average	85		5.9	4.5 (0.64)	3.9 (0.65)

**Table 3:** Summary of I-TASSER modeling on the Benchmark III

			C_α_-RMSD (Å) (TM-score) of I-TASSER models	
			
Protein name	Length (residues)	Secondary structure	First cluster	Best in top five clusters
1ne3A	56	β	4.6 (0.45)	4.6 (0.48)
2cr7A	60	α	4.5 (0.48)	2.6 (0.66)
2f3nA	65	α	1.8 (0.74)	1.8 (0.74)
1itpA	68	αβ	10.9 (0.33)	4.5 (0.40)
1kviA	68	αβ	2.0 (0.72)	2.0 (0.72)
1b4bA	71	αβ	6.4 (0.48)	5.6 (0.54)
1gjxA	77	β	6.9 (0.44)	5.6 (0.47)
1of9A	77	α	3.6 (0.53)	3.6 (0.53)
1mn8A	84	α	7.0 (0.35)	7.0 (0.35)
1fo5A	85	αβ	3.8 (0.54)	3.8 (0.54)
1ten_	87	β	1.6 (0.85)	1.6 (0.85)
1fadA	92	α	3.6 (0.59)	3.6 (0.59)
1no5A	93	αβ	10.6 (0.43)	10.5 (0.45)
1g1cA	98	β	2.5 (0.79)	2.5 (0.79)
1cqkA	101	β	1.5 (0.88)	1.5 (0.88)
1abv_	103	α	13.0 (0.28)	6.8 (0.40)
1jnuA	104	αβ	2.7 (0.75)	2.7 (0.75)
1egxA	115	αβ	2.3 (0.80)	2.3 (0.83)
1gyvA	117	β	3.3 (0.78)	3.3 (0.78)
1orgA	118	α	2.4(0.78)	2.4(0.78)
Average	87		4.8 (0.60)	3.9 (0.63)
